# Improving survival of acute-on-chronic liver failure patients complicated with invasive pulmonary aspergillosis

**DOI:** 10.1038/s41598-018-19320-2

**Published:** 2018-01-17

**Authors:** Jie Gao, Qing Zhang, Yuankui Wu, Ying Li, Tingting Qi, Congyan Zhu, Sijia Liu, Ruoxi Yu, Qinjun He, Weiqun Wen, Fuyuan Zhou, Yongpeng Chen, Jinjun Chen, Jinlin Hou

**Affiliations:** 1 Hepatology Unit, Department of Infectious Diseases, Nanfang Hospital, Southern Medical University, Guangzhou, China; 2 Pharmacy Department, Nanfang Hospital, Southern Medical University, Guangzhou, China; 3 Department of Medical Imaging, Nanfang Hospital, Southern Medical University, Guangzhou, China; 4 Internal Medicine, Puning People’s Hospital, Puning, China

## Abstract

The mortality of acute-on-chronic liver failure (ACLF) patients complicated with invasive pulmonary aspergillosis (IPA) was extremely high. We aimed to explore prognostic value of the Chronic Liver Failure-Sequential Organ Failure Assessment (CLIF-SOFA) lung score and to establish an optimal voriconazole regimen for ACLF patients complicated with IPA. We retrospectively screened hospitalized ACLF patients in our hospital from July 2011 to April 2016, from which 20 probable IPA cases were diagnosed. Along with onsets of IPA, deteriorated diseases severity, especially lung conditions were found in those 20 ACLF patients. It was found that IPA patients with CLIF-SOFA lung score <2 had better 28-day survival than those with lung score >1 (11/13 vs 0/7, *p* < 0.001). Based on plasma voriconazole concentration measurement, an optimal voriconazole regimen (loading doses: 0.2 g twice daily; maintenance doses, 0.1 g once daily) was established, which resulted in rational trough plasma drug concentrations (1–5 μg/mL), good clinical outcomes (90-day survival rate of 6/8) and no observed adverse events. In conclusion, CLIF-SOFA lung score >1 was able to identify ACLF patients complicated with IPA encountering much higher 28-day mortality. An optimal voriconazole regimen was safe and effective in our ACLF patients complicated with IPA.

## Introduction

Acute-on-chronic liver failure (ACLF) is a distinct clinical entity characterized by acute deterioration of liver function, multi-organ failure and high mortality^1,2^. Infections, including spontaneous bacterial peritonitis and pulmonary infection, are consistently attributed to the development and progression of ACLF^1,3^.

Moreover, some fungal infections, especially invasive pulmonary aspergillosis (IPA), are observed in patients with decompensated cirrhosis, liver failure or severe alcoholic hepatitis^4–6^. Patients with critical liver disorders are now considered as additional risk factors^7^, along with the classic factors such as allogeneic bone marrow transplantation^8^.

The prevalence of IPA in HBV-related ACLF patients has been reported to be 5.0–8.3%^9–11^, reaching 13.8% (13/94) in patients with severe alcoholic hepatitis^6^. The short-term mortality observed in these patients ranged from 73.5% to 100%^6,9,10^. However, currently no criteria are available to identify patients with poor prognosis.

Voriconazole, liposomal amphotericin B and echinocandins are three frequently prescribed drugs for IPA patients, among which voriconazole is recommended as the first-line option for primary treatment of IPA^12^. However, use of oral or intravenous voriconazole in ACLF patients has been limited due to its potential hepatotoxicity, and also the lack of pharmacokinetics or pharmaco-dynamics data in such critical situations^13^. Additionally, acute kidney injuries related to voriconazole treatment is believed to be related with sulfobutylether-β-cyclodextrin in the intravenous formulation of voriconazole^14^, and treatment with oral instead of intravenous voriconazole should eliminate any risk of acute kidney injury related to sulfobutylether-β-cyclodextrin.

Management strategies for IPA in ACLF patients have evolved significantly in our Hepatology Unit since 2012, from which an intensive IPA screening strategy was implemented following physicians’ consistent awareness of IPA in liver failure patients. And since plasma voriconazole monitoring methods became available in our hospital in 2014, we explored an optimal voriconazole regimen for ACLF patients complicated with IPA. Our retrospective study aimed to prove the association between CLIF-SOFA lung score^1^ and short-term outcomes, and to introduce an optimal voriconazole regimen for ACLF patients complicated with IPA.

## Results

### IPA cohort derived from ACLF patients

**Figure 1:**
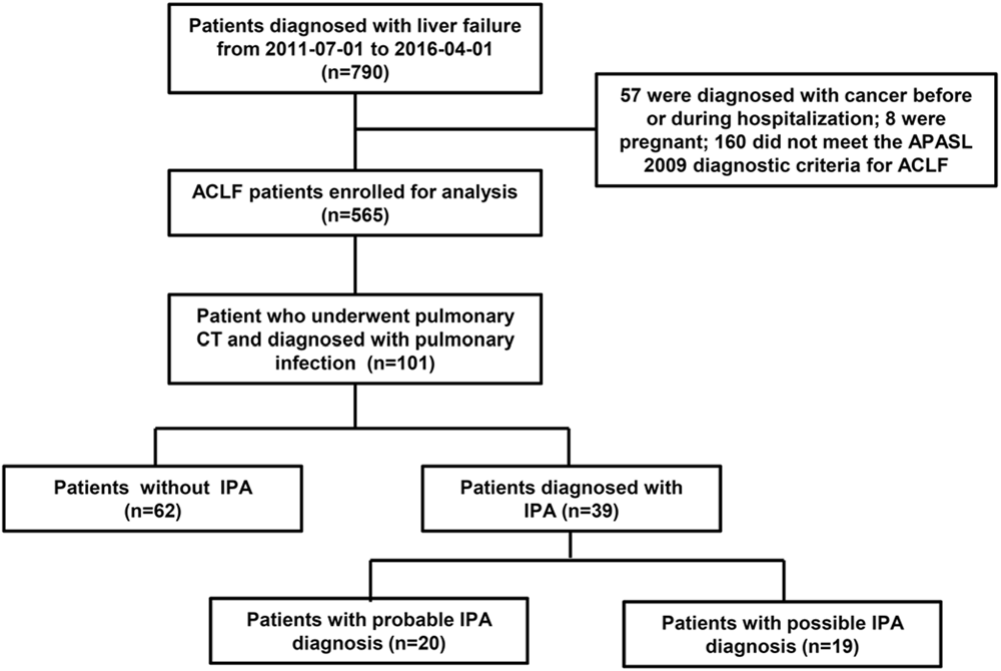
Flow diagram of patients screening.

In total, 790 adult patients who had been diagnosed with liver failure in the Hepatology Unit of Nanfang Hospital between July 2011 and April 2016 were screened and 565 ACLF cases were included, among which 101 ACLF patients who underwent pulmonary CT scanning and were diagnosed with lung infection were re-evaluated (Fig. 1).

Thirty-nine patients were identified as potential IPA cases with corresponding radiological features, among which 18 plasma samples from 16 patients were available for galactomannan (GM) tests. Positive GM were obtained in 7/10 plasma samples collected on the second day of voriconazole treatment, in 4/6 plasma samples collected 1–2 days prior to anti-fungal treatment, and in 0/2 samples collected 5–6 days prior to anti-fungal treatment. Overall 11 patients had positive GM tests (median GM index: 1.89, ranging from 1.07 to 3.21).

Appropriate sputum cultures were performed in 27 cases, and 51.9% (14/27) cases were identified as positive for *Aspergillus* spp. (13 with *A. fumigatus* and 1 with *A. Flavus*). Overall, 20 probable IPA were established by positive culture of *Aspergillus* from respiratory secretions (n = 9), positive plasma GM tests (n = 6) or both (n = 5).

In order to avoid potential bias from the possible diagnosed IPA, the following analysis was focused on the 20 probable IPA patients. The main underlying causes of the ACLF patients with probable IPA were HBV-related liver disease (19/20). The median time from ACLF diagnosis to IPA development was 13 days, ranging from 0–44 days. Supplementary Table 1 showed the detailed radiologic features of 20 probable IPA patients. Concomitant bacterial lung infections occurred in three IPA patients (1 *Staphylococcus haemolyticus*, 1 *Enterobacter cloacae*, 1 *Klebsiella pneumoniae*). One IPA patients complicated with bacterimia, and three IPA cases complicated with spontaneous bacterial peritonitis.

We enrolled 62 ACLF patients diagnosed with non-*aspergillus* lung infection and compared their characteristics with those 20 probable IPA patients (Table 1). Corticosteroids were prescribed more frequently in ACLF patients who developed IPA (60% vs 11.3%, *p* < 0.001), and the cumulative prednisone-equivalent dosages were also higher (240 mg vs 70 mg, p = 0.045) than those with non-*aspergillus* lung infection. IPA developments were not attributed to severity of liver failures, as indicated by comparable MELD, MELD-Na, CLIF-C ACLFs and CLIF-SOFA scores^15^ between these two groups (Table 1).

**Table 1:** The characteristics of ACLF patients without IPA, and with development probable IPA at enrolment or at IPA diagnosis.

Characteristics	IPA development (−), n = 62	IPA development (+), n = 20		*p*-value
		At enrolment	At IPA diagnosis	
Age, (years)	44 [22, 83]	42 [26, 70]		
Male, (n, %)	55 (88.7)	18 (90.0)		
Other sites infection, (n, %)	5 (8.0)	4 (21.1)		
Receipt of corticosteroids, (n, %)	7 (11.3)^**^	12 (60.0)		
Cumulative prednisone-equivalent dose, (mg)^a^	70 [30, 360]^*^	240 [38, 800]		
Temperature, (°C)	36.5 [36.0, 37.5]^**^	37 [36.3, 39.6]	38.6 [37, 39.6]	0.001
C- reactive protein, (mg/L)	12.5 [0.4, 87.1]	13.5 [1.1, 99.8]	34.9 [3.0, 69.4]	0.084
Procalcitonin, (ng/mL)	0.8 [0.1, 39.7]	0.8 [0.4, 6.3]	1.0 [0.2, 3.1]	0.878
Leukocyte count, (10^9^/L)	7.1 [1.6, 31.0]^*^	9.4 [3.7, 34.7]	15.0 [3.6, 34.7]	0.148
Platelet count, (10^9^/L)	90 [66, 210]	113.5 [11, 199]	54 [24, 199]	0.001
Serum bilirubin, (mg/dL)	22.9 [6.4, 46.8]	25.5 [8.1, 43.7]	28.4 [9.7, 47.3]	0.028
Aspartate aminotransferase, (U/L)	163.5 [29.0, 2136.0]	174.5 [32.0, 3000.0]	112.0 [32.0, 279.3]	0.031
Alanine aminotransferase, (U/L)	118.5 [17.0, 3163.0]	83.3 [15.0, 2084.1]	75.6 [15.0, 197.2]	0.047
International normalized ratio	2.2 [1.5, 4.7]	2.2 [1.5, 4.1]	2.5 [1.5, 4.6]	0.234
Albumin, (mg/L)	29.9 [20.0, 41.0]	33.7 [20.2, 40.4]	34.7 [20.9, 40.3]	0.649
Creatinine, (mg/L)	0.8 [0.3, 5.5]	0.8 [0.4, 2.8]	0.7 [0.4, 2.8]	0.683
Serum sodium, (mmol/L)	137 [123, 148]	135 [125, 143]	134 [123, 140]	0.105
CLIF-SOFA^1^	8 [6, 11]	8 [6, 13]	10 [6, 15]	0.083
Cerebral failure (n, %)	2 (3.2)	1 (5.0)	3 (15.0)	0.605
Liver failure (n, %)	52 (83.9)	16 (80.0)	19 (95.0)	0.342
Lung failure (n, %)	0 (0)	0 (0)	3 (15.0)	0.231
Coagulation failure (n, %)	22 (35.5)	9 (45.0)	11 (55.0)	0.752
Kidney failure (n, %)	5 (8.1)	2 (10.0)	2 (10.0)	1
Circulation failure (n, %)	0 (0)	0 (0)	0 (0)	—
CLIF-C ACLFs^15^	39.7 [25.6, 58.2]	44.9 [29.9, 57.1]	46.8 [29.4, 72.6]	0.028
MELD^15^	26.4 [8.9, 40.1]	24.0 [13.6, 38.5]	27.2 [15.4, 40.9]	0.145
MELD-Na^15^	27.8 [12.4, 57.3]	25.7 [15.2, 52.8]	28.6 [15.4, 60.0]	0.064

(*p < 0.05, **p < 0.01, compared with IPA patients at enrolment. ^a^Patients received corticosteroids were compared).

### IPA worsened overall conditions of ACLF patients

To figure out the clinical feature of ACLF patients complicated with IPA, we analyzed the changes in clinical and laboratory index in ACLF patients as IPA developed, compared with those at enrolment (Table 1).

The temperature elevated as IPA developed (median: 37°C vs 38.6°C, *p* = 0.001). Some infection index such as C-reactive protein, procalcitonin and Leukocyte count didn’t change significantly as IPA developed. IPA also exacerbated the overall condition of ACLF patients, as indicated by higher CLIF-C ACLFs score (median: 44.9 vs 46.8, *p* = 0.028) and trends for higher MELD-Na (median: 25.7 vs 28.6, *p* = 0.064) and CLIF-SOFA score (median: 8 vs 10, *p* = 0.083). In detail, the numbers of lung failure (0 to 3), cerebral failure (1 to 3), liver failure (16 to 19) and coagulation failure (9 to 11) increased as IPA developed, although they were not statistically different (Table 1).

### CLIF-SOFA lung score >1 at IPA diagnosis was associated with higher mortality

Among the 20 probable IPA patients, 11 died within 90 days. Most of the deaths were caused by lung failure associated with IPA (9/11), the other two deaths were caused by intra-cerebral and digestive tract hemorrhage, respectively.

**Figure 2:**
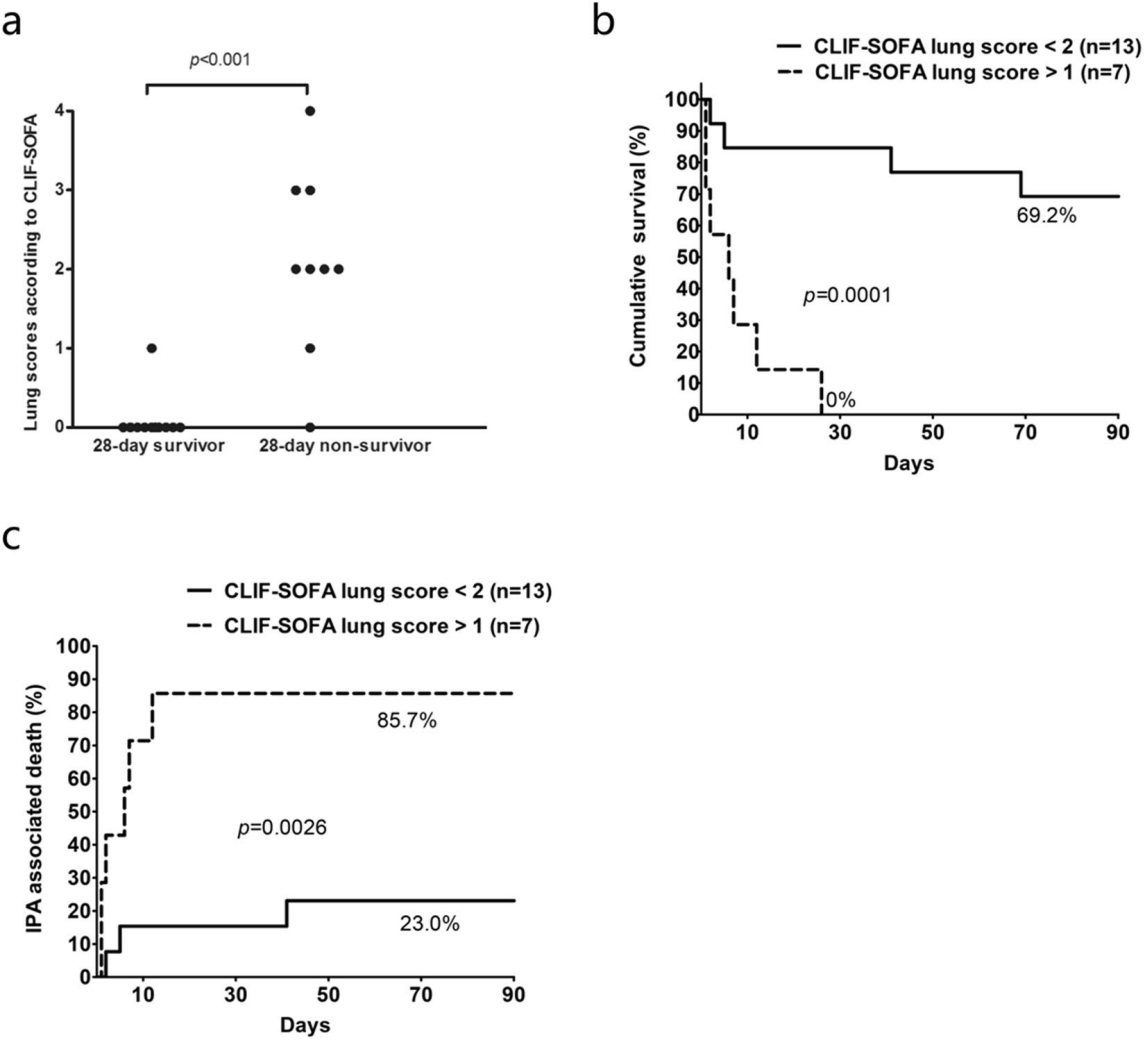
CLIF-SOFA lung scores were able to differentiate IPA patients with poor prognosis. (**a**) CLIF-SOFA scores were greater in 28-day non-survivors than survivors. (**b**) Higher lung was associated with poorer overall survival and (**c**) more IPA-associated death.

Patients with poor 28-day outcome had a significantly worsen lung condition at the time of IPA diagnosis, as shown by higher CLIF-SOFA lung scores (Fig. 2a). Further analysis showed that lung score was associated with death within 28-days in ACLF patients complicated with IPA (RR: 2.803, 95% CI: 1.543–5.091, *p* = 0.001). Lung score with cut-off at 1, which was defined with maximally selected log-rank statistic (M = 3.4256, *p* < 0.001), was adopted as the index to identify patients with high mortality. The 28-day (0% vs 84.6%, *p* < 0.001) and 90-day (0% vs 69.2%, *p *< 0.001, Fig. 2b) survival rates calculated from the time of IPA diagnosis were much poorer in patients with lung score >1 (n = 7) than those <2 (n = 13). Consistent results were found with respect to 28-day and 90-day IPA-associated mortality rates (Fig. 2c).

Between patients with CLIF-SOFA lung score >1 and those <2, the median time interval (days) from ACLF diagnosis to IPA diagnosis was comparable [18 (0–44) vs 12 (0–33), *p* = 0.780]. All (7/7) patients with lung score >2 had fever as IPA diagnosed and for cases with lung score <1 the number was 11/13, *p* = 0.521. The median time interval (days) from fever to IPA diagnosis was also comparable [6.5 (2–14) vs 4.0 (1–14), *p* = 0.237] between patients with lung score >1 and those with lung score <2.

Probable IPA patients with lung score >1 had more severe conditions than those <2, as determined by higher CURB-65, PSI^16^, MELD, MELD-Na, CLIF-C ACLFs and CLIF-SOFA scores. Infection index such as C-reactive protein, procalcitonin and leukocyte count was comparable between these two groups.

### Optimization of the voriconazole regimen

Voriconazole is a first-line option for primary treatment of IPA^12^, however experience of its use in ACLF patients is limited due to concerns of potential liver injuries. In our previous clinical practice, several ACLF patients with a prompt IPA diagnosis were treated with voriconazole using the recommended protocol for Child-Pugh A or B liver cirrhosis. The doses always had to be adjusted due to overt drug-related adverse effects. Thus, we explored to optimize the voriconazole regimen in ACLF patients since plasma voriconazole concentrations monitoring methods became available in our hospital (in October 2014).

The detail of the process was shown in the Supplementary material: Voriconazole monitoring protocol. Briefly, we optimized the loading doses of voriconazole based on ideal trough plasma concentrations (1–5 µg/mL) measured on day 1 and optimized maintenance doses based on plasma concentrations on days 3–5. In total, 10 IPA patients (8 probable and 2 possible) underwent plasma voriconazole concentration monitoring.

**Figure 3:**
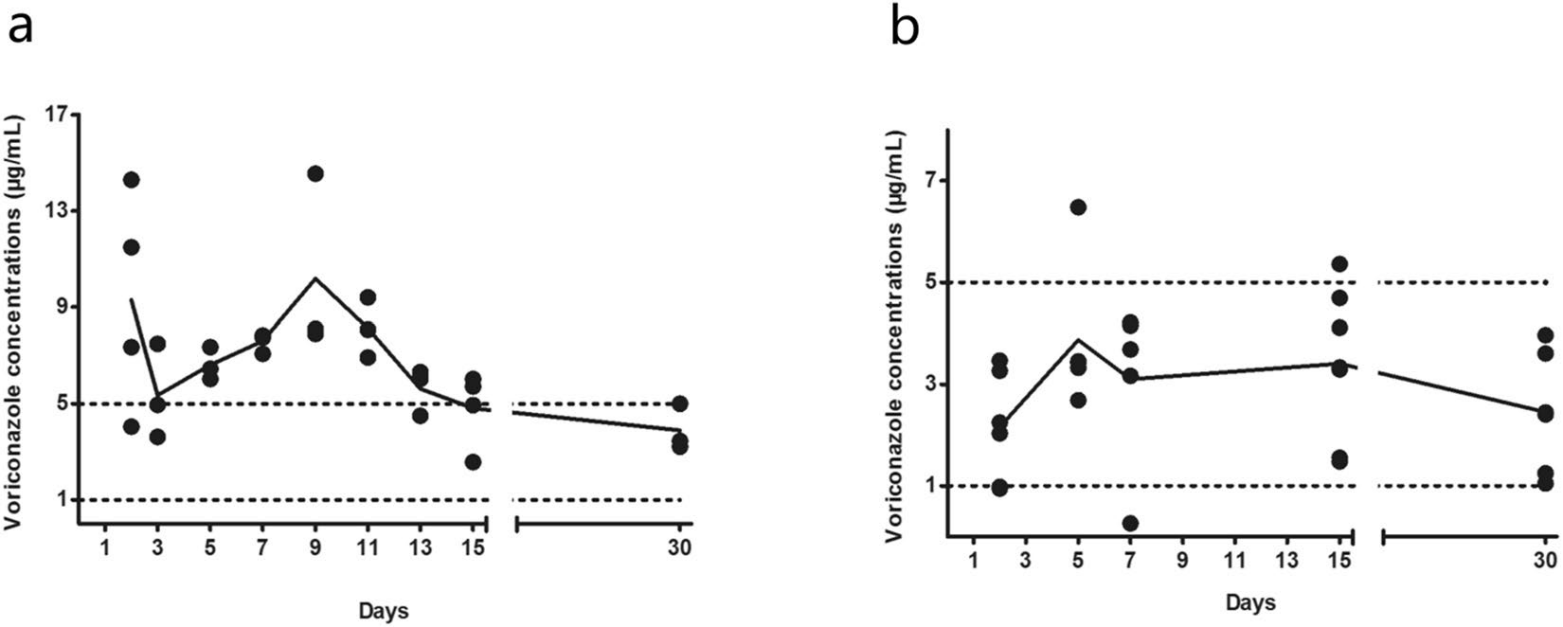
The blood voriconazole concentrations in ACLF patients complicated with IPA. (**a**) Patients started with the standard dosage regimen and underwent dosage adjustments based on the plasma voriconazole levels (n = 4). (**b**) Patients received the optimal dosage regimen de novo (n = 6).

The first four patients began with the dose of the standard regimen (loading dose, 0.4 g, po, q12h; maintenance dose, 0.2 g, po, q12h) and underwent dosage adjustment based on the plasma voriconazole levels. The standard regimen usually produced extremely high plasma drug concentrations (Fig. 3a), much further exceeding the efficient therapeutic ranges (1–5 µg/mL). Two of them even complicated with severe adverse events (visual disturbance), and had to stop voriconzole for about half a mouth awaiting for the voriconazole concentrations falling to rational ranges. Finally, after several adjustments based on plasma voriconazole levels, the maintenance doses were set at 0.1 g/d for two (body weight: 65 Kg, 44 Kg), 0.2 g/d for one (body weight: 99 Kg), 75 miligrams for one (body weight: 65 Kg).

Thus, in the subsequent 6 patients (median body weight with range: 64 [62–71] Kg), the loading dose was set at 0.2 g, po, q12h; in one patient, we give a maintenance dose of 0.2 g/d, which was finally reduced to 0.1 g/d because of elevated plasma voriconazole level (6.48 µg/mL); the other 5 patients received maintenance dose of 0.1 g/d de novo, which resulted in stable and optimal trough voriconazole concentrations (1–5 µg/mL, Fig. 3b). No observed adverse events were recorded in these 6 patients.

Finally, the optimal voriconazole regimen was established (loading dose, 0.2 g, po, q12h; maintenance dose, 0.1 g, po, qd), which achieved optimal therapeutic plasma drug concentrations.

**Figure 4:**
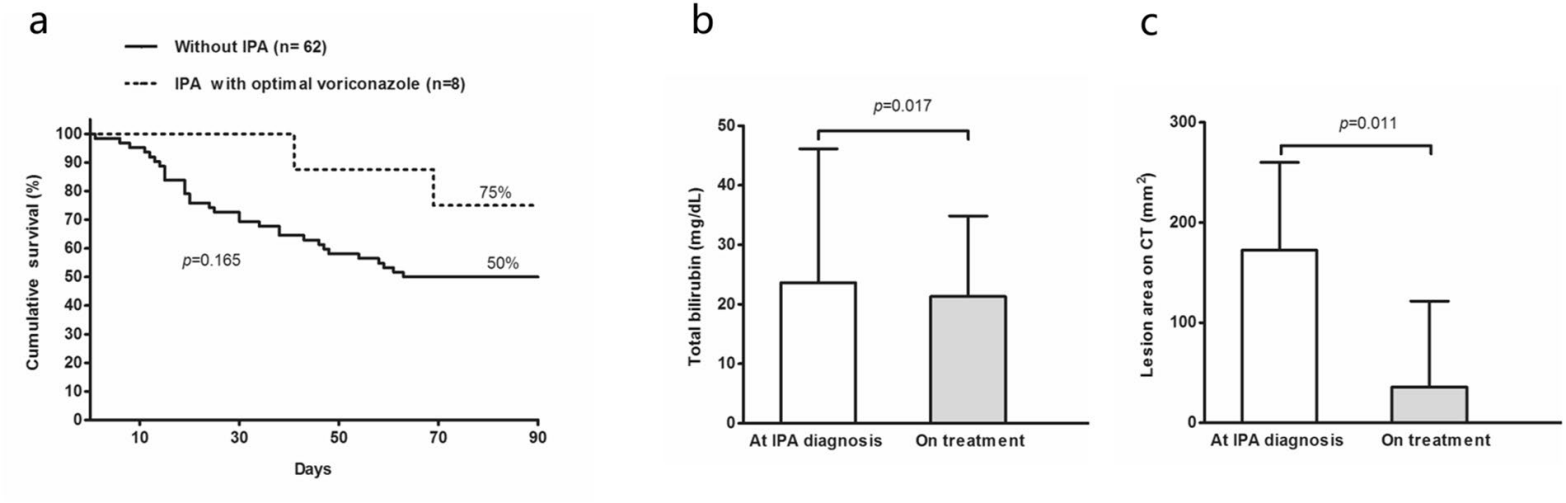
Clinical outcomes in IPA patients treated with optimal voriconazole regimen. (**a**) IPA Patients treated with optimal voriconazole regimen had similar 90-day survival as patients without IPA. (**b**) Bilirubin level (median with range) reduced with one weeks of optimal voriconazole regimen treatment. (**c**) Lesions on pulmonary CT scans (median with interquartile range) resolved obviously with 1–2 weeks of voriconazole treatment.

Probable IPA patients treated with optimal voriconazole regimen (n = 8) achieved a comparable 90-day survival to ACLF patients without IPA in our cohort (Fig. 4a). Also, an obvious reduction in total bilirubin (median: 23.6 mg/dl vs 20.8 mg/dl, *p* = 0.017) or resolution of pulmonary lesions on thoracic CT (median:172.33 mm^2^ vs 35.97 mm^2^, *p* = 0.011) was observed in these patients (Fig. 4b,c).

The median treatment duration of the optimal voriconazole regimen was 54 days (14–110), which was similar to those recommended for the standard one in non-ACLF patients^12^. However, the cumulative Defined Daily Dose (cDDD) reduced dramatically to 15.25 (4–40).

## Discussion

The present study provides several conclusions with clinical merits. Firstly, we found that IPA exacerbated organ failures in ACLF patients, especially lung failures. Secondly, we confirmed the prognostic values of CLIF-SOFA lung scores in ACLF patients complicated with probable IPA. Finally, we introduced an optimal voriconazole regimen, which resulted in rational trough plasma drug concentrations (1–5 μg/mL), no observed adverse events and good anti-fungal responses.

As the progression of IPA, despite of the overall deteriorated condition, lung dysfunction was the most predominant and distinct symptom in IPA patients, and lung failure was the crucial cause of death in these patients. These findings indicated the important role of lung condition at the time of IPA diagnosis in predicting the therapeutic efficiency and outcomes in these patients.

Subsequently, we proved that CLIF-SOFA lung score >1 at IPA diagnosis was able to identify patients with poor prognosis. Patients with lung score >1 at IPA diagnosis had poor responses to anti-fungal treatments and high short-term mortality, which indicated that lung score >1 might represent a delayed diagnosis of IPA, regardless of variable IPA progressions due to different host and pathogenic factors. Thus, early diagnosis of IPA could have a determined role in improve the outcome in ACLF patients. This idea was in accordance with other studies. Wang *et al*.^11^ and Gustot *et al*.^6^ reported that in the context of HBV-ACLF or severe alcoholic hepatitis, patients with IPA ceased mainly due to respiratory failure within a very short time of IPA diagnosis, regardless of various anti-fungal therapies. Both reports suggested the vital importance of a prompt IPA diagnosis^6,11^.

However, due to non-specific clinical manifestations and lack of conscious on IPA in ACLF patients, early diagnosis has remained a challenge. Since September 2012, we implemented an IPA screening strategy according to the AspICU study group^17^. Thus, ACLF patients were screened with thoracic CT driven by clinical symptoms and signs, such as fever refractory to at least 3 days of broad-sputum antibiotic therapy, pleuritic chest pain, pleuritic rub, dyspnoea, haemoptysis. The percentage of patients encountered higher CLIF-SOFA lung scores decreased these years. Except for this symptom-driven screening strategy, further studies on time-triggered or overall condition-triggered screening strategy, including low-radiation doses thoracic CT and plasma GM tests in high-risk ACLF patients were warranted for early diagnosis furthermore^18^. And CLIF-SOFA lung score <2 had potential as a surrogate marker defining early IPA diagnosis, albeit further validations were warranted in future studies.

We summarized the reports about IPA in patients with advanced liver diseases and found the overall high short-term mortality (>70%, Table 2). We realized that in addition to difficulty of prompt diagnosis, the other challenging issue was the treatment options of IPA in ACLF patients. The first-line treatment is voriconazole, but this drug is potentially hepatotoxic and contraindicated because of hepatic metabolism^13^. Using therapeutic drug monitoring methods, we established an optimal voriconazole regimen in ACLF patients. Our voriconazole regimen was able to maintain stable and rational therapeutic trough concentrations between 1 and 5 µg/mL^19^. Meanwhile, patients treated with optimal voriconazole regimen had good clinical outcomes and 90-days survival rate high as 75%, which was also benefitted from early IPA diagnosis as indicated by lower CLIF-SOFA lung score (<2) in all patients prior to our optimal regimen prescribed. Thus, the anti-fungal efficacy of our optimal voriconazole regimen on ACLF patients with higher CLIF-SOFA lung score was unclear and needed prospective observations.

**Table 2:** IPA in non-liver transplant patients with critical liver diseases. **(**CSID: Chinese Society for Infectious Diseases. CSH: Chinese Society for Hepatology. CSCC: Chinese Society for Critical Care. Cas: Caspofungin. Vor: Voriconazole).

Author (year)	Ref	liver diseases	ACLF criteria	IPA criteria	IPA Incidence	Transplant-free Mortality	Anti-fungal treatments Cas/Vor/others
Wang, 2010	11	HBV ACLF	CSID/CSH	EORTC/MSG	66/798 (8.3%)	100% (30 days)	C43/V0/0
Wu, 2012	10	HBV ACLF	APASL	EORTC/MSG	29/470 (6.1%)	86.2% (in hospital)	C0/V29/0
Chen, 2013	9	HBV ACLF	APASL	EORTC/MSG	39/787 (5%)	94.8% (90 days)	C10/V9/20 others
Gustot, 2014	6	Alcoholic hepatitis	—	EORTC/MSG	13/94 (13.8%)	100% (40 days)	C6/V2/6 others
Falcone, 2011	5	Critical liver disorders	—	EORTC/MSG	—	48/67 (71.6%)	C13/V5/30 others
Zhao, 2011	28	HBV ACLF	CSID/CSH	CSCC 2007	83/681 (12.1%)	73.49% (in hospital)	C0/V0/others
Zou, 2012	29	HBV ACLF	CSID/CSH	EORTC/MSG	39/967 (4%)	79.5% (30 days)	C6/V0/others
Liu, 2013	30	HBV ACLF	CSID/CSH	EORTC/MSG	20/463 (4.3%)	75% (in hospital)	C0/V5/others

The dosage of oral voriconazole in our optimal regimen for ACLF patients was much lower than that recommended for patients without liver disease or with Child-Pugh A/B liver cirrhosis^12,19^. Voriconazole undergoes extensive hepatic metabolism by cytochrome P450 system enzymes, including CYP2C19, 2C9 and 3A4^19^. Patients with liver failure may have a diminished capacity to metabolize the drug, leading to its accumulation and hazardous plasma concentrations. Drug interactions in the cytochrome P450 system may also influence the plasma concentration of voriconazole^20,21^. For example, proton pump inhibitors, especially omeprazole which was commonly used in our ACLF patients, have a potential role in elevating plasma voriconazole levels^22^. Our optimal voriconazole regimen was derived from Chinese ACLF patients, who had moderate body weights and CYP2C19 polymorphism for slow voriconazole metabolism. Thus, its feasibility in other ethnicities who differ in CYP2C19 polymorphisms and body weight need further validation.

Our study has several limitations that are mainly related to its retrospective nature. Because there were no lung biopsies due to coagulation disorders in liver failure patients and no autopsies for the deceased patients, we did not establish the proven IPA diagnosis. It was a single-centre study and the sample size was small. Our optimal voriconazole needed further validations in future studies, which should be multi-centered, prospective, large sized and ideally multi-ethnicity ones.

Our study showed for the first time that CLIF-SOFA lung score was a valuable prognostic index in ACLF patients complicated with IPA, and our optimal voriconazole regimen was safe and effective in those critical ill patients.

## Patients and Methods

### Patients with ACLF

A retrospective chart review was conducted to identify ACLF patients with a documented clinical diagnosis of IPA between July 2011 and April 2016. Patients diagnosed with liver failure during hospitalization (Hepatology Unit, Department of Infectious Diseases, Nanfang Hospital) were screened (Supplementary Table 1 showed the ICD-10 code for liver failure). ACLF diagnosis was reassessed according to the Asian Pacific Association for the Study of the Liver (APASL) 2009 consensus recommendations for ACLF^23^. Patients who were diagnosed with a malignant tumour or who were found pregnant before or during hospitalization were excluded from the study.

### Diagnosis of IPA

#### Radiological evaluations

Among the included ACLF patients, those underwent pulmonary computed tomography (CT; Philips Brilliance 256-slice spiral CT) scans after enrolment and showed signs of lung infection were reassessed by Dr Wu and Dr Yu (Supplementary Table 2 showed the ICD-10 code for lung infection). For patients with suspected IPA features, the numbers, sizes and locations (based on the pulmonary segments) of pulmonary lesions such as nodules or masses shown on CT images prior to and after 1–2 week anti-fungal treatment were measured and recorded.

#### Mycological criteria

Among the included ACLF patients, sputum culture reports from the clinical laboratory were reviewed to identify patients positive for *Aspergillus* (*A*.) spp. In addition, in patients showed suspicious IPA image manifestation, available cryogenic plasmas, which had been stored at −20 °C for 6 months to 4 years, were tested for galactomannan (GM) following the manufacturer’s instructions (EIA, Bio-Rad Laboratories) with a cut-off value of 0.5^24^.

#### Diagnostic criteria of IPA and non-*aspergillus* lung infection

The IPA diagnosis was established according to the European Organization for Research and Treatment of Cancer/Mycoses Study Group (EORTC/MSG) definitions, considering ACLF as a moderate risk factor for IPA (Supplementary Table 3)^17,25^. In detail, ACLF patients must meet with the radiologic features of IPA (dense, well-circumscribed lesions with or without a halo sign, aircrescent sign or cavity, masses), and the mycological criteria: direct test (direct microscopy, or culture) indicating the presence of *Aspergillus* species in appropriate lower-respiratory tract sputum or galactomannan antigen detected in plasma.

Non-*aspergillus* lung infection was established by the combination of clinical symptoms and new infiltration on pulmonary CT^26^, with probable or possible IPA excluded.

### IPA treatment

Anti-fungal therapies for ACLF patients complicated with IPA have evolved in our Hepatology Unit. In the first few years (2011–2014), our patients were treated with echinocandins (Cancidas, caspofungin acetate, Merck) or standard voriconazole (voriconazole tablet, 50 milligrams/tablet; Huashen, Chendu, PR China) dosage (loading dose as 0.4 g, po, q12h, maintenance dose as 0.2 g, po, q12h, which was adjusted mainly based on overt clinical adverse effects).

In October 2014, plasma voriconazole concentrations monitoring tests became available in our hospital. We then began to explore an optimal voriconazole regimen based on the plasma drug level (Supplementary material: Voriconazole monitoring protocol). Finally, an optimal voriconazole regimen was established, which consisted of a lower loading dose (0.2 g, po, q12h) and a lower maintenance dose (0.1 g, po, qd), and this optimal regimen turned to be the preferred anti-fungal option for the majority of the subsequent IPA cases with underlying ACLF.

### Data collection and outcome evaluation

Clinical data including history, physical examination, laboratory measurements, imaging findings and treatment strategies were recorded. Outcomes were evaluated based on the medical records or by direct contact with patient or a kin. Total bilirubin changes were evaluated after around one week of anti-fungal treatment. CT lesions changes were evaluated 1–2 weeks since anti-fungal therapies started. We documented overall 28-day and 90-day survivals and IPA-associated deaths. IPA-associated death was defined as death related to respiratory failure as alternative causes of death excluded, which was restricted in patients with no sustained favourable response to anti-fungal treatment as evaluated with pulmonary CT changes and clinical symptoms and signs^27^. Informed consent had been obtained from all patients and/or their relatives about usage of their clinical data and/or samples. This study was approved by the Ethics Committee of Nanfang Hospital, Southern Medical University (NFEC-201105-K1). Methods were carried out in accordance with the approved guidelines.

### Statistical analysis

Data were presented as the median (range) for continuous variables. Fisher’s exact test, the Wilcoxon test, or the Mann-Whitney U test was performed as appropriate. The Cox model was used to test the association between lung scores and short-term mortality in IPA patients. Patient survival rates were assessed using the Kaplan-Meier method and compared with the log-rank test. All above mentioned statistical analyses were performed using SPSS software (IBM SPSS, version 17.0). Differentiation of delayed from prompt IPA diagnosis using CLIF-SOFA lung score was determined with maximally selected log-rank statistic (R statistical software, version 3.4.0). All tests were two sided with a significance level of 0.05.

### Availability of materials and data

The datasets generated during and/or analysed during the current study are available from the corresponding author on reasonable request.

## Electronic supplementary material

Supplementary information

Supplementary tables
