# Supplementary material for: Improving survival of acute-on-chronic liver failure patients complicated with invasive pulmonary aspergillosis
Source: Sci Rep. 2018 Jan 17;8:876. doi: 10.1038/s41598-018-19320-2 (PMC5772638; doi:10.1038/s41598-018-19320-2)
Supplement: Supplementary file 1 — Supplementary information [file 41598_2018_19320_MOESM1_ESM.pdf]

Improving survival of acute-on-chronic liver failure patients complicated with invasive pulmonary aspergillosis

Jie Gao[1*], Qing Zhang[2*], Yuankui Wu[3*], Ying Li[1,4], Tingting Qi[1], Congyan Zhu[1], Sijia Liu[2], Ruoxi Yu[3], Qinjun He[1], Weiqun Wen[1], Fuyuan Zhou[1], Yongpeng Chen[1], Jinjun Chen[1], Jinlin Hou[1]

[1]Hepatology Unit, Department of Infectious Diseases, Nanfang Hospital, Southern Medical University, Guangzhou, China

[2]Pharmacy Department, Nanfang Hospital, Southern Medical University, Guangzhou, China

[3]Department of Medical Imaging, Nanfang Hospital, Southern Medical University, Guangzhou, China

[4]Internal Medicine, Puning People's Hospital, Puning, China

*These authors contributed equally to this article

Corresponding author

Dr Jinjun Chen

Hepatology Unit, Department of Infectious Diseases, Nanfang Hospital, Southern Medical University, Guangzhou, China

Phone: 86+18588531001 Fax: 862062787423 Email: chjj@smu.edu.cn

Protocol for plasma voriconazole concentration monitoring guided treatment for IPA patients with advanced liver disease

Background ;

The prevalence of IPA in Chinese ACLF patients is reported to be 4-5% with a high mortality rate ranged from 70% to 100%. Voriconazole is first line option for IPA therapy, but lack of pharmacokinetics or pharmacodynamics in ACLF patients and the frequently associated adverse events hinders its use in clinical practice. Plasma voriconazole monitoring methods was introduced into Nanfang hospital since 2013.

Aim :

1. To figure out the pharmacokinetic features of voriconazole in IPA patients with advanced liver disease.
2. To establish an optimal voriconazole dosage regimen in patients with advanced liver disease.

Methods :

Patients

Patients with decompensated cirrhosis or ACLF were under close supervision for IPA. Once IPA was suspected, patients were enrolled for study. Concerned about safety, patients with the following characteristics were excluded: with type 1 hepatorenal

symdrome, with edema encephalopathy, under blood dialysis, MELD>35, under Ventilator assisted ventilation. The decompensated cirrhosis was defined by the acute development of large ascites, hepaticencephalopathy, gastrointestinal hemorrhage or bacterial infections, or any combination of these on patients with previous established or un-established cirrhosis. ACLF was diagnosed according to the APASL 2009 diagnostic criteria for ACLF. Briefly, acute hepatic insult manifesting as jaundice (serum bilirubin≥5 mg/dl [85umol/l]) and coagulopathy (INR ≥1.5 or prothrombin activity<40%) , complicated within 4 weeks by ascites and/or encephalopathy in a patient with previously diagnosed or undiagnosed chronic liver disease. IPA patients were identified according to the revised European Organization for Research and Treatment of Cancer/Mycoses Study Group (EORTC/MSG) definitions. In brief, proven IPA was characterized by documented histopathological and microbiological evidence of aspergillus spp. infection, either in autopsy or in biopsied tissue or culture samples from a normally sterile site. Probable IPA was characterized by the presence of radiological (nodules, cavities, halos or air crescent signs on CT) and microbiological (direct microscopy, culture, Galactomannan(GM) test) features in a patient with appropriate host factors. Possible IPA was characterized by the presence of respiratory symptoms and radiological features in patients with host factors. Advanced liver disease was added to the host factors in our study.

Procedures:

Patients were treated with oral voriconazole (loading dose:_400mg twice on day1,

maintenance dose :200mg, twice daily(BID) from the day2 on).The plasma voriconazole concentration was measured from day 2th to day7th, day 10th, day 20th, day 30th, or depended on the voriconazole level and dosage changing.

The loading dose was adjusted according to the drug concentration on day2. If the drug level on day2 were above 5ug/ml for most patients, the loading dose should be reduced according to certain levels. Otherwise, loading dose should be increased or stay unchanged.

The maintenance dose was adjusted according to the drug concentration after day2. For patients the trough blood voriconazole level between 1-5ug/ml, a repeated trough voriconazole level was obtained on d10 d20 d30 respectively. For patients trough voriconazole level<1ug/ml, the maintenance dose should be increased according to the drug level. For patients trough voriconazole level>5ug/ml, stop administration and obtained the trough Vor level the next days. The drug should not restart until the trough Vor level<5ug/ml and the dose should be decreased by according to the certain level.

Samples and Measurements

The plasma samples (2ml for each person one time) should be collected with anticoagulation tubes and stored at 4℃ at most for 24 hours before tests. LC-MS/MS (Agilent 6460) method was employed for plasma voriconazole concentration measurement.
