# Supplementary material for: Improving survival of acute-on-chronic liver failure patients complicated with invasive pulmonary aspergillosis
Source: Sci Rep. 2018 Jan 17;8:876. doi: 10.1038/s41598-018-19320-2 (PMC5772638; doi:10.1038/s41598-018-19320-2)
Supplement: Supplementary file 2 — Supplementary tables [file 41598_2018_19320_MOESM2_ESM.pdf]

Improving survival of acute-on-chronic liver failure patients complicated with invasive pulmonary aspergillosis

Jie Gao[1*], Qing Zhang[2*], Yuankui Wu[3*], Ying Li[1,4], Tingting Qi[1], Congyan Zhu[1], Sijia Liu[2], Ruoxi Yu[3], Qinjun He[1], Weiqun Wen[1], Fuyuan Zhou[1], Yongpeng Chen[1], Jinjun Chen[1], Jinlin Hou[1]

[1]Hepatology Unit, Department of Infectious Diseases, Nanfang Hospital, Southern Medical University, Guangzhou, China

[2]Pharmacy Department, Nanfang Hospital, Southern Medical University, Guangzhou, China

[3]Department of Medical Imaging, Nanfang Hospital, Southern Medical University, Guangzhou, China

[4]Internal Medicine, Puning People's Hospital, Puning, China

*These authors contributed equally to this article

Corresponding author

Dr Jinjun Chen

Hepatology Unit, Department of Infectious Diseases, Nanfang Hospital, Southern Medical University, Guangzhou, China

Phone: 86+18588531001 Fax: 862062787423 Email: chjj@smu.edu.cn

Supplementary tables

Table s1. Demographic, clinical and biochemical characteristics of patients at the time of IPA diagnosis according to CLIF-SOFA lungs core.

**Characteristic**	**Higher lung score (>1) (n=7)**	**Lower lung score (<2) (n=13)**	***p* value**
Age (years)	41 [31, 70]	43 [26, 58]	0.578
Male (n, %)	6 (85.7)	12 (93.2)	1
Time from enrolment to IPA diagnosis (days)	18 [0, 33]	12 [0, 44]	0.780
Time from fever to IPA diagnosis (days)*	6.5 [2.0, 14.0]	4.0 [1.0, 14.0]	0.237
Fever (n, %)	7 (100)	11 (84.6)	0.521
Other infection (n, %)	1 (14.2)	3 (23.1)	1
Voriconazole treatment (n, %)	3 (42.9)	11 (84.6)	0.122
Radiological features			
Well-shaped nodule(s) with holo sign (n,%)	0 (0)	3 (23.1)	0.521
Well-shaped nodule(s) without holo sign (n,%)	5 (71.4)	10 (76.9)	1
Air-crescent sign (n,%)	0/7	0/13	-
Cavitation (n,%)	1 (14.3)	0 (0)	0.350
Masses/consolidation (n,%)	6 (85.7)	3 (23.1)	0.017
Pleural fluid (n,%)	2 (28.6)	5 (38.5)	0.526
Bilateral lung infection (n,%)	7 (100)	11 (84.6)	0.521
C-reactive protein (mg/L)	30.0 [16.0, 52.6]	35.3 [3.0, 69.4]	1.000
Procalcitonin (ng/mL)	1.3 [0.9, 1.9]	1.0 [0.2, 3.1]	0.524
Leukocyte count (109/L)	16.8 [6.1, 23.0]	15.0 [3.6, 34.7]	0.861
Platelet count (109/L)	57 [27, 100]	54 [24, 199]	0.693
Total bilirubin (mg/dL)	28.8 [9.7, 47.3]	24.9 [16.7, 46.1]	0.285
Aspartate aminotransferase (U/L)	175.9 [35.0, 209.0]	87.5 [32.0, 279.3]	0.108
Alanine aminotransferase (U/L)	75.6 [49.5, 194.0]	74.7 [15.0, 197.2]	0.735
International normalized ratio	2.8 [1.8, 4.6]	2.4 [1.5, 3.5]	0.104
Albumin (mg/L)	25.4 [22.7, 37.4]	34.8 [20.9, 40.3]	0.446
Creatinine (mg/L)	1 [0.5, 2.8]	0.7 [0.4, 2.2]	0.122
Serum sodium (mmol/L)	128 [123, 139]	135 [130, 140]	0.032
PSI[16]	109 [81，169]	83 [46，118]	0.005
CURB-65[16]	1 [0，3]	1 [0，2]	0.097
MELD [15]	33.3 [18.4, 40.9]	23.8 [15.4, 37.6]	0.052
MELD-Na [15]	37.7 [25.1, 60.0]	27.6 [15.4, 45.6]	0.008
CLIF-C ACLFs [15]	44.18 [29.4, 57.1]	50.53 [47.0, 72.6]	0.008
CLIF-SOFA [1]	12 [10, 15]	8 [6, 11]	0.002
Cerebral failure (n, %)	3 (42.9)	0 (0)	0.031
Liver failure (n, %)	6 (85.7)	13 (100)	0.350
Lung failure (n, %)	3 (42.9)	0 (0)	0.031
Coagulation failure (n, %)	5 (71.4)	6 (46.2)	0.374
Kidney failure (n, %)	1 (14.3)	1 (7.7)	1
Circulation failure (n, %)	0 (0)	0 (0)	-

Data are expressed as the median [min-max] for continuous variables and counts (percentage) for categorical variables.

MELD: Model for end-stage liver disease.

CLIF-SOFA: Chronic Liver Failure-Sequential Organ Failure Assessment.

*Patients with fever before diagnosis are compared.

Table s2. ICD-10 codes for related diseases for patient screening.

Disease	ICD-10 code
**Related to liver failure**	
Acute alcoholic hepatic failure	K70.401
Acute drug induced hepatic failure	K71.102
Acute hepatic failure	K72.000
Acute liver function failure	K72.003
Sub-acute drug-induced hepatic failure	K71.104
Sub-acute hepatic failure	K72.001
Chronic hepatic failure	K72.100
Chronic alcoholic hepatic failure	K70.402
Hepatic failure, nospecified	K72.900
**Related to lung infections**	
Lung infection	J98.414
Pneumonia	J18.900
Pulmonary fungal infection	B49. x14
Pulmonary aspergillosis	B44.100+J99.8*

Note: Patients diagnosed with "Acute-on-chronic liver failure" without ICD-10 code records were also screened, since acute-on-chronic liver failure has no related ICD-10 code yet.

Table s3. The adapted diagnostic criteria for IA based on EORTC/MSG and AspICU criteria.

Probable IA (all three criteria must be met)

- Host risk factor: ACLF is considered a risk factor
- Clinical features: radiological features (dense, well-circumscribed lesion (s) with or without a halo sign, aircrescent sign or cavity, mass(es))
- Mycological criteria: direct test (cytology, direct microscopy, or culture) indicating the presence of *Aspergillus* species or galactomannan antigen detected in plasma

Possible IA

- Presence of host factor and radiological features (dense, well-circumscribed lesion (s) with or without a halo sign, aircrescent sign and cavity, masses), but in the absence of mycological criteria
